# Drug-Resistant Tuberculosis among Children, China, 2006–2015

**DOI:** 10.3201/eid2311.170234

**Published:** 2017-11

**Authors:** Ning-ning Tao, Xiao-chun He, Xian-xin Zhang, Yao Liu, Chun-bao Yu, Huai-chen Li

**Affiliations:** Shandong Provincial Hospital affiliated to Shandong University, Jinan, China (N.-N. Tao, Y. Liu, H.-C. Li);; Baoji Central Hospital, Baoji, China (X.-C. He);; Shandong Provincial Chest Hospital, Jinan (X.-X. Zhang, C.-B. Yu)

**Keywords:** pediatric tuberculosis, drug-resistant tuberculosis, primary transmission, epidemiology, tuberculosis and other mycobacteria, children, China

## Abstract

Microbial drug resistance has become a major public health concern worldwide. To acquire epidemiologic data on drug-resistant tuberculosis (DR TB) among children, a major cause of illness and death for this population, we conducted a retrospective study of 2006–2015 data from 36 TB prevention and control institutions in Shandong Province, China. A total of 14,223 new TB cases, among which children (<18 years of age) accounted for only 5.5%, were caused by culture-confirmed *Mycobacterium tuberculosis*. Among children with TB, 18.9% had DR TB and 6.9% had multidrug-resistant TB. Over the past decade, the percentage of DR TB; multidrug-resistant TB; and overall first-line drug resistance for isoniazid, rifampin, ethambutol, and streptomycin among children increased significantly (at least 12%). Understanding the long-term trends of DR TB among children can shed light on the performance of TB control programs, thereby contributing to global TB control.

Tuberculosis (TB) is one of the leading causes of death worldwide (*1*). The World Health Organization (WHO) estimates that worldwide during 2015, this disease developed in 10.4 million persons and caused the death of 1.8 million (*2*). Children <15 years of age account for an estimated 10% of those affected by the global TB burden, corresponding to 1 million cases. Each year, ≈0.2 million children die of TB, which means that 2 children die of this disease every 5 minutes (*2*).

The attention given to TB in children has increased since the 2006 publication of the first edition of Guidance for National Tuberculosis Programmes on the Management of Tuberculosis in Children (*3*). However, expanding prevalence of TB globally, especially drug-resistant (DR) TB among children, is still a major cause of childhood illness and death (*4*). Control of TB among children is impeded by the challenges of presentation, diagnosis, reporting, and treatment; the absence of clear targets; and perceptions of low public health importance of TB (*5*). Difficulties with sputum collection and the paucibacillary nature of TB in children often make TB diagnosis difficult and drug-susceptibility testing (DST) inaccessible because DST is possible only after bacteriologic confirmation (*4**–**7*). Accordingly, children initially receive treatment for drug-susceptible TB (*7*). Because of its paucibacillary nature, childhood TB has been deemed less infectious and neglected by TB prevention and control institutions (*4**–**6*). Even some diagnosed and treated TB cases in children failed to be recorded in registers or reported to national TB programs (*8*).

Children with TB are especially susceptible to severe disease and death (*9**–**12*); even those with a favorable treatment outcome (cure or completion) or a latent infection could become a reservoir of disease relapse or reactivation (*13*). Although children metabolize drugs more rapidly than adults, guidance on drug regimens, dosages, appropriate monitoring, and duration of therapy for children is frequently extrapolated from adult data (*14*). With few drug options and limited experience, treatment for children with DR TB is complex. TB in children represents recent transmission and can be considered a sentinel of disease spreading throughout the community (*13**,**15*). 

In this study, we compared baseline characteristics for children (<18 years of age) and adults (>18 years of age) in Shandong Province, China, who had new cases of TB during 2006–2015. From a longitudinal perspective, we comprehensively assessed the burden of DR TB patterns among children. 

## Materials and Methods

### Ethics

Ethics approval was obtained from the Ethics Committee of Shandong Provincial Hospital, affiliated with Shandong University, Shandong, China. Before analysis, patient records were anonymized and deidentified.

### Study Population and Data Collection

This retrospective cohort study was conducted among 36 monitoring sites: 13 municipal-level local health departments, 21 county-level hospitals, and 2 province-level hospitals (Shandong Provincial Hospital and Shandong Provincial Chest Hospital). Monitoring site selection was based on convenience and reflection of a range of TB burdens and clinical capacities. New culture-confirmed TB cases that occurred in Shandong Province during 2006–2015 were consecutively collected from the China Information System for Disease Control and Prevention (http://www.chinacdc.cn/). In 2004, the Center for TB Control and Prevention of Shandong Province established the Katharine Hsu International Research Center of Human Infectious Diseases, the provincial health department where trained researchers collected and recorded patient information on a standard case report form. Since then, the Katharine Hsu Center has been responsible for laboratory quality assurance and TB surveillance in Shandong Province.

*Mycobacterium tuberculosis* was identified by culture; susceptibility to isoniazid, rifampin, ethambutol, and streptomycin was identified by DST. Information for all patients (age, sex, TB contact history, disease sites [pulmonary and extrapulmonary], and prior TB treatment history) was collected and recorded.

### Laboratory Methods

Pulmonary samples were collected by expectoration, gastric aspiration, and sputum induction. Extrapulmonary samples (pleural fluid, spinal fluid, and lymph nodes) were collected by pleural tap, lumbar puncture, lymph node biopsy, fine needle aspiration, and other techniques (*3*).

All samples available from suspected sites of involvement were processed for smear and culture. Tissue samples were also examined for the presence of granulomas. To identify the presence of acid-fast bacilli, we used Ziehl-Neelsen staining for smear microscopy. Each sample was cultured on Lowenstein-Jensen culture medium. *M. tuberculosis* was identified according to combined growth characteristics, morphologic characteristics of the colony, and inhibition by p-nitrobenzoic acid (*16*). Samples containing nontuberculous mycobacteria were eliminated.

DST was performed by using the proportion method on Lowenstein-Jensen medium and the following drug concentrations: isoniazid (0.2 μg/mL), rifampin (40 μg/mL), ethambutol (2.0 μg /mL), and streptomycin (4.0 μg/mL) (*17*). Isolates with growth proportion for >1% on medium containing anti-TB drugs compared with the growth on drug-free medium were considered to be resistant to those drugs.

### Laboratory Quality Control

The National Tuberculosis Reference Laboratory of the Chinese Center for Disease Control and Prevention and the Supranational Tuberculosis Reference Laboratory of the Public Health Laboratory Hong Kong were responsible for external quality assessment (EQA) (*16*). EQA for smear, culture, and DST in county- and district-level laboratories was conducted by the prefectural and provincial TB laboratories; EQA in the provincial reference laboratories and the National Tuberculosis Reference Laboratory was conducted by the Supranational Tuberculosis Reference Laboratory according to WHO guidelines (*17*). Blinded retesting of a random selection of ≈10% of isolates from each laboratory by a superior laboratory was essential.

### Data Inclusion and Definitions

We included all patients with new TB cases and a positive *M. tuberculosis* culture for whom DST results, demographic information, and clinical information were obtainable. We excluded patients with nontuberculous mycobacteria infection and patients with HIV co-infection (in China, HIV-positive patients are immediately transferred to HIV-specialized hospitals).

Childhood TB was defined as TB in a patient <18 years of age. A TB isolate susceptible to all 4 of the tested first-line drugs was defined as drug-susceptible. MDR TB was defined as TB resistant to at least isoniazid and rifampin. TB contact was defined as contact with family members or schoolmates with TB long enough to enable long-term exposure (*18*). Bilateral disease means bilateral lesions (such as the tree-in-bud sign, bronchiectasis, cavitary pulmonary disease, and other inflammation signs) on radiologic images. A patient who had received anti-TB treatment for <1 month was classified as a new TB case-patient; a patient who had received anti-TB treatment for >1 month was classified as a previously treated TB case-patient (*17*).

### Statistical Analyses

We analyzed the changes in proportions of the different resistance patterns over time by using the χ^2^ test for trends and linear regression. From univariable analyses we obtained odds ratios (ORs) and 95% CIs, for comparison of specific characteristics between child and adult TB case-patients by Pearson χ^2^ test. p<0.05 was considered to be significant. Statistical analyses were performed by using SPSS software, version 17.0 (SPSS Inc., Chicago, IL, USA) (*19*).

## Results

### Characteristics of Patients

We analyzed demographic, clinical, and laboratory information for 14,223 new TB case-patients in Shandong Province who had had culture-confirmed *M. tuberculosis* infection during the past decade. The mean ± SD age of these patients was 43.3 ± 19.6 years, and 784 (5.5%) patients were <18 years of age. Of the 784 children with TB, 597 (76.1%) were >15 years of age, 101 (12.9%) were >13 but <15 years of age, 86 (11.0%) were <13 years of age, and only 32 (4.0%) were <5 years of age.

Adults with TB were more likely than children with TB to be male (OR 2.13, 95% CI 1.84–2.44) and to have cavitary pulmonary disease according to chest radiographs (OR 1.38, 95% CI 1.17–1.61) (Table 1). Children with TB were more likely to have extrapulmonary disease (OR 0.83, 95% CI 0.70–1.00) and to have had contact with a TB case-patient (OR 0.59, 95% CI 0.42–0.83) who was a family member or schoolmate.

**Table 1:** Sociodemographic and clinical characteristics of child and adult TB patients, Shandong Province, China, 2006–2015*

Characteristics	Age <18 y, no. (%), n = 784	Age >18 y, no. (%), n = 13,439	Total OR (95% CI), n = 14,223	p value	
Male sex	458 (58.42)	10,078 (74.99)	2.134 (1.842–2.437)	<0.001	
Extrapulmonary TB	154/784 (19.64)	2,274/13,439 (16.92)	0.833 (0.695–0.999)	0.05	
TB contact†	39/784 (4.97)	396/13,134 (3.02)	0.594 (0.424–0.832)	0.002	
Chest radiology					
Cavitary pulmonary disease	220/779 (28.24)	4,683/13,334 (35.12)	1.375 (1.172–1.614)	<0.001	
Bilateral disease‡	218/401 (54.36)	2,929/5,051 (57.99)	1.159 (0.945–1.421)	0.16	

*OR, odds ratio; TB, tuberculosis. †Contact with family members or schoolmates with TB long enough for long-term exposure (*18*). ‡Bilateral lesions such as “tree-in-bud,” bronchiectasis, cavitary pulmonary disease, and other inflammation signs seen on radiologic images.

### Drug-Resistance Patterns

Among isolates from 784 new TB case-patients <18 years of age, the highest proportion of resistance was found for streptomycin (14.3%), followed by isoniazid (12.1%), rifampin (8.3%), and ethambutol (5.5%). MDR TB was found in 54 (6.9%) of these 784 children. Resistance to all 4 tested first-line drugs was found for 32 (59.3%) of the children with MDR TB. Among patients <18 years of age, 148 (18.9%) had cases resistant to >1 first-line drug and 52 (6.6%) had cases resistant to either isoniazid or rifampin (but not both). The proportion of overall ethambutol resistance and resistance to all 4 tested first-line drugs was significantly higher among children than adults (p = 0.001). The proportion of resistance to isoniazid or rifampin (but not both) was significantly lower among children than adults (p = 0.03) (Table 2).

**Table 2:** First-line drug resistance found for 14,233 new cases of TB, Shandong Province, China, 2006–2015*

Drug resistance	<18 y, no. (%), n = 784	>18 y, no. (%), n = 13,439	p value
Any resistance to first-line drug	148 (18.88)	2,853 (21.23)	0.12
INH	95 (12.12)	1,884 (14.02)	0.14
RIF	65 (8.29)	1,112 (8.27)	0.99
EMB	43 (5.48)	449 (3.34)	0.001
SM	112 (14.29)	2,084 (15.51)	0.36
Resistance to 1 drug	67 (8.55)	1,323 (9.84)	0.23
INH	20 (2.55)	445 (3.31)	0.25
RIF	5 (0.64)	129 (0.96)	0.36
EMB	3 (0.38)	33 (0.25)	0.45
SM	39 (4.97)	716 (5.33)	0.67
Resistance to 2 drugs	27 (3.44)	683 (5.08)	0.04
INH + RIF	3 (0.38)	113 (0.84)	0.22
INH + EMB	2 (0.26)	30 (0.22)	0.70
INH + SM	17 (2.17)	459 (3.42)	0.06
RIF + EMB	1 (0.13)	4 (0.03)	0.25
RIF + SM	4 (0.51)	74 (0.55)	1.00
SM + EMB	0	3 (0.02)	NA
Resistance to 3 drugs	22 (2.81)	548 (4.08)	0.08
INH + RIF + EMB	2 (0.26)	15 (0.11)	0.24
INH + RIF + SM	17 (2.17)	468 (3.48)	0.05
INH + EMB + SM	2 (0.26)	55 (0.41)	0.77
RIF + EMB + SM	1 (0.13)	10 (0.07)	0.46
Resistance to at least INH/RIF	52 (6.63)	1,206 (8.97)	0.03
Multidrug resistant, overall	54 (6.88)	895 (6.66)	0.80
Resistance to 4 drugs	32 (4.08)	299 (2.22)	0.001

*EMB, ethambutol; INH, isoniazid; NA, not applicable; RIF, rifampin; SM, streptomycin; TB, tuberculosis.

### Trends over Time

**Figure 1:**
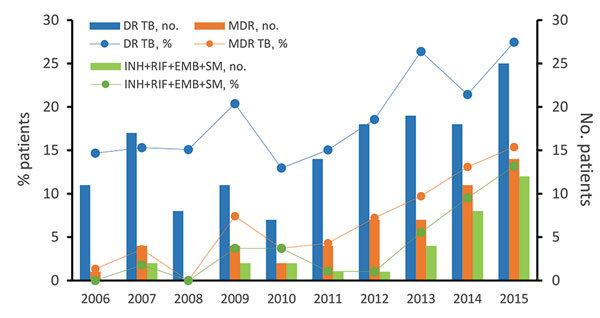
Trends for DR TB and MDR TB among children with primary cases of TB, Shandong Province, China, 2006–2015. The χ^2^ and linear regression results are shown in Table 3. DR TB, drug-resistant TB; EMB, ethambutol; INH, isoniazid; MDR, multidrug-resistant; RIF, rifampin; SM, streptomycin; TB, tuberculosis.

**Figure 2:**
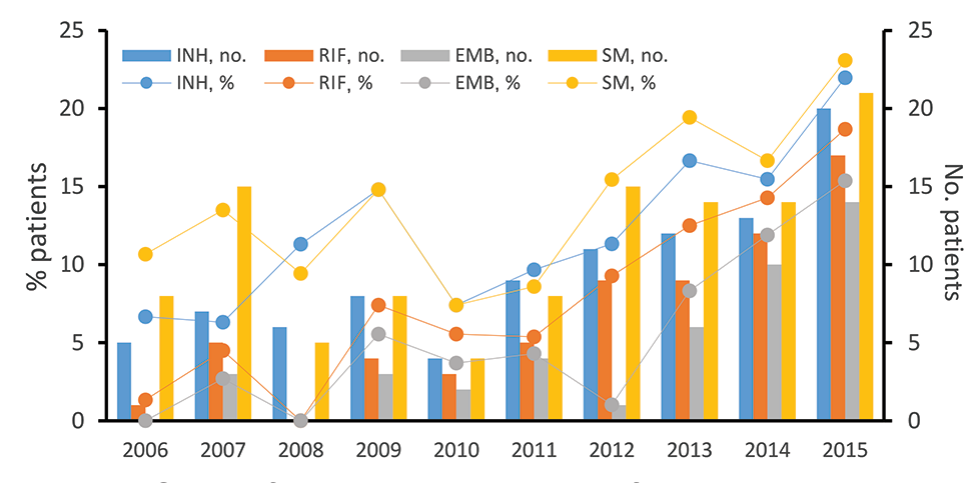
Overall first-line drug resistance for INH, RIF, EMB, and SM in primary cases of tuberculosis in children, Shandong Province, China, 2006–2015. The χ^2^ and linear regression results are shown in Table 3. EMB, ethambutol; INH, isoniazid; RIF, rifampin; SM, streptomycin.

Among the 784 new cases of TB in children, the proportion that were DR TB increased from 14.7% in 2006 to 27.5% in 2015, a yearly increase of 1.3% (R^2^ = 0.58; χ^2^ test for trends: χ^2^ = 7.231, p = 0.007). Over the past decade, MDR TB increased yearly at a rate of 1.5% (R^2^ = 0.79; χ^2^ test for trends: χ^2^ = 21.916, p<0.001), from 1.3% to 15.4%. The percentage of a special type of MDR TB (resistance to all 4 tested first-line drugs) also increased 1.2% per year (R^2^ = 0.64; χ^2^ test for trends: χ^2^ = 22.836, p<0.001), from 0.0% to 13.2% (Figure 1). In addition, over the past decade, the estimated percentage of overall first-line drug resistance for isoniazid, rifampin, ethambutol, and streptomycin increased significantly (p<0.001 for isoniazid, rifampin, and ethambutol; p = 0.01 for streptomycin) (χ^2^ = 12.879, for isoniazid resistance, increasing at a yearly rate of 1.3% [R^2^ = 0.64] from 6.7% to 22.0%; χ^2^ = 26.743 for rifampin resistance, increasing at a yearly rate of 1.8% [R^2^ = 0.84] from 1.3% to 18.7%; χ^2^ = 24.972 for ethambutol resistance, increasing at a yearly rate of 1.4% [R^2^ = 0.68] from 0.0% to 15.4%; χ^2^ = 6.555 for streptomycin resistance, increasing at a yearly rate of 1.1% [R^2^ = 0.47] from 10.7% to 23.1%) (Table 3, Figure 2).

**Table 3:** Changes in proportions of different *Mycobacterium tuberculosis* resistance patterns, Shandong Province, China, 2006–2015*

Resistance pattern	χ^2^	p value	R^2^	X-coefficient	SE
Drug-resistant TB	7.231	0.007	0.58	0.013	0.117
Resistant to INH	12.879	<0.001	0.64	0.013	0.048
Resistant to RIF	26.743	<0.001	0.84	0.018	−0.019
Resistant to EMB	24.972	<0.001	0.68	0.014	−0.024
Resistant to SM	6.555	0.01	0.47	0.011	0.077
Multidrug resistance	21.916	<0.001	0.79	0.015	−0.015
Resistant to INH + RIF + EMB + SM	22.836	<0.001	0.64	0.012	−0.024

*EMB, ethambutol; INH, isoniazid; MDR, multidrug-resistant; RIF, rifampin; ; SM, streptomycin; TB, tuberculosis.

## Discussion

This 10-year retrospective cohort review of children with TB in the second largest province of China describes the clinical characteristics of TB in children and the epidemiology of DR TB among children. The major findings of this study are as follows: 1) among 784 new, treated cases of TB, an estimated 5.5% were in patients <18 years of age, among which children <15 years accounted for only 23.9%; 2) children with new cases of TB were more likely than adults to have extrapulmonary TB or a history of contact with TB patients; 3) 18.9% of TB cases in children were DR TB and 6.9% were MDR TB (over half of which were resistant to all 4 drugs tested; and 4) over the past decade, the percentage of DR TB, MDR TB, and overall first-line drug resistance among children has increased significantly.

The low sensitivity (*20*) of microbiological testing among children (sputum smear microscopy <5%, sputum culture 15%) may exacerbate the discrepancies between the number of expected cases and reported cases (*4**–**6*). Culture-confirmed TB in children <15 years of age accounts for only 1.3% of new TB cases in this study, far lower than the predicted proportion of childhood TB in China (>5%) (*5*). According to WHO data, TB incidence among young children <5 years of age was predicted to make up 58% (interquartile range 40–77) of that among total patients <15 years (*5*). Underreporting was more pronounced for the younger age group worldwide and especially in China (*5*). Young children <5 years of age accounted for only 4% of all TB cases in children during this study period. Children, especially young children <5 years of age, often have paucibacillary disease, and obtaining specimens is difficult, which prevents microbiological diagnosis (*21**,**22*), a vital element for patient selection in this study. The diagnosis of TB in children should be made cautiously by experts after thorough assessment of all evidence derived from a careful history, clinical examination, bacteriological confirmation, and relevant investigations (*3*). Unfortunately, in most low- and middle-income countries, the recommended contact investigations, including TB contact tracing for children suspected of having disease and contact screening for young children living close to a source case-patient, were rarely and inconsistently conducted (*18*). Without effective contact investigation, TB, especially DR TB, in children is rarely diagnosed and treated, which may worsen the situation. Although risks for severe disease and death are highest among children, TB in young children is the least likely to be confirmed bacteriologically (*20*). All these factors together suggest that effective diagnostic methods to microbiologically confirm TB, and regular contact investigations are urgently needed to refine future estimates of the incidence of TB and DR TB among children in China.

According to the most recent national DR TB survey in China, the reported proportions of new cases of DR TB and MDR TB were 34.2% and 5.7%, respectively (*16*); the proportions of new cases of DR TB and MDR TB among children in our study were 18.9% and 6.9%, respectively. Because drug resistance rarely develops for children during treatment (*23*), the high proportion of primary cases of MDR TB in children in our study may reflect recent transmission of MDR TB strains in Shandong. Previous surveys reported that patients who had a history of prior contact with a TB patient were more likely to have MDR TB (*24**,**25*). In this study, more contact with TB was recorded among children than adults (p = 0.002). Because of the lack of standardized protocols for the therapy of childhood DR TB, children are empirically given the few formulations that are available for children and based on DST results, which are hard to access and often delayed (*26**,**27*). The fact that more than half of the new cases of MDR TB in children in this study (59.3%, 32/54) were resistant to all 4 tested first-line anti-TB drugs made the situation much worse. Reducing community-transmitted drug resistance and basing therapy on each patient’s (children and source case-patients) DST results may enlighten future childhood TB control strategies (*6**,**28*).

The percentage of DR TB, MDR TB, and overall first-line drug resistance for isoniazid, rifampin, ethambutol, and streptomycin in primary cases of TB in children increased significantly over the study period. This finding indicates ongoing primary transmission of DR TB strains in China. Ongoing primary transmission of DR TB strains among children may cause catastrophic consequences. Other studies have reported that independent host factors that predispose to TB recurrence are malnutrition, smoking, HIV infection, and other immunosuppressive states (*29*). After the state of the host changes, even a person with a favorable treatment outcome (cure or completion) or a latent infection could become a reservoir for disease relapse or reactivation (*30*). To make things worse, the strongest risk factor for acquired DR and the highest risk for death is retreatment, as has occurred in Limpopo (South Africa) (*31*), Uganda (*32*), and Malaysia (*33*). 

This study had some limitations. First, because we examined only 1 province on the eastern coast of China, the economic and regional disparities limited the generalizability of the results. Second, because we included only children with culture-confirmed TB, we did not analyze those who were treated on the basis of DR TB contact history or who had poor clinical response to therapy. Third, in this retrospective study, medical records provided little information on source cases, education, and living conditions; consequently, we failed to show the relationships between these factors and the DR TB epidemic. Last, the lack of genotyping (the standard for identifying the origin of resistant isolates) impeded us from correlating the mutations in the observed strains with the source strains in the environment in which the children lived.

In conclusion, primary cases of DR TB in children in Shandong Province, China, increased over the past decade. DR TB strains, especially MDR TB, are mainly transmitted by airborne infection from an adult source case-patient (*3*). To control the ongoing primary transmission of DR TB among children, especially among children in close contact with patients with diagnosed TB, more effective strategies are urgently needed. For more individualized anti-TB regimens for children, DST should be performed for both first- and second-line anti-TB drugs among children and their sources; regular contact investigations should also be performed. Moreover, ongoing reforms for financing TB diagnosis and treatment for children will be essential components of effective interventions for TB prevention and control in China. 

If the global TB control strategy continues to pay less attention to the usually asymptomatic, paucibacillary, noncontagious childhood TB (*22*), the goal of achieving zero deaths from childhood TB by 2025 will be difficult to reach (*34*); control of TB in children and adults still faces huge challenges. Understanding the long-term trends of DR TB among children can shed light on the performance of TB control programs in China, thereby contributing to global TB control.
